# What musicians do to induce the sensation of groove in simple and complex melodies, and how listeners perceive it

**DOI:** 10.3389/fpsyg.2014.00894

**Published:** 2014-08-15

**Authors:** Guy Madison, George Sioros

**Affiliations:** ^1^Department of Psychology, Umeå UniversityUmeå, Sweden; ^2^Sound and Music Computing Group, INESC TECPorto, Portugal

**Keywords:** groove, music, musicians, movement, rhythm, syncopation, micro-timing

## Abstract

Groove is the experience of wanting to move when hearing music, such as snapping fingers or tapping feet. This is a central aspect of much music, in particular of music intended for dancing. While previous research has found considerable consistency in ratings of groove across individuals, it remains unclear how groove is induced, that is, what are the physical properties of the acoustic signal that differ between more and less groove-inducing versions. Here, we examined this issue with a performance experiment, in which four musicians performed six simple and six complex melodies in two conditions with the intention of minimizing and maximizing groove. Analyses of rhythmical and temporal properties from the performances demonstrated some general effects. For example, more groove was associated with more notes on faster metrical levels and syncopation, and less groove was associated with deadpan timing and destruction of the regular pulse. We did not observe that deviations from the metrical grid [i.e., micro-timing (MT)] were a predictor of groove. A listener experiment confirmed that the musicians' manipulations had the intended effects on the experience of groove. A Brunswikian lens model was applied, which estimates the performer-perceiver communication across the two experiments. It showed that the communication achievement for simple melodies was 0.62, and that the matching of performers' and listeners' use of nine rhythmical cues was 0.83. For complex melodies with an already high level of groove, the corresponding values were 0.39 and 0.34, showing that it was much more difficult to “take out” groove from musical structures designed to induce groove.

## Introduction

Groove is a central aspect of music appreciation, closely connected to functional uses of music such as dance, drill, and ritual. Given that these behaviors involve synchronization and coordination, which relies on a common temporal reference, it seems likely that the temporal properties of this signal and its representation might be crucial to the understanding of groove (Merker et al., 2009; Madison et al., 2011). For the purposes of the present study, groove is defined as “the sensation of wanting to move some part of your body in relation to some aspect of the music” Madison, 2006). For comparison, Janata et al. (2012) asked participants within a synchronization experiment to provide descriptions of their experience in their own words. Based on frequently occurring words they arrived at the definition that: “groove is the aspect of the music that induces a pleasant sense of wanting to move along with the music.” There is a remarkable similarity between the theoretically derived definition proposed by Madison (2006) and the crowd-sourced definition of Janata et al. (2012).

Listeners exhibit substantial agreement in their ratings of groove, even when they have widely varying musical experience and are presented with unfamiliar music (Madison, 2006). This suggests that some physical properties in the audio signal have universal effects on the experience of groove. Beat tempo seems to have little effect in and of itself, as it has been shown that each music performance is optimized for the tempo it is played in (Madison and Paulin, 2010), and tempo is only weakly related to groove (Madison, 2003, 2006; Madison et al., 2011). Madison et al. (2011) measured a number of higher-order rhythmic properties in the sound signals of 100 commercially available music recordings, and related them to listeners' ratings of groove for each recording. The results indicated that physical correlates of the experience of groove might include (1) beat salience. i.e., the number and loudness of sounds that occur on the beat, (2) event density, i.e., the number and loudness of sound events per unit time, (3) fast metrical levels, i.e., the metrical subdivisions that sound events are articulated in, and (4) systematic micro-timing (MT), i.e., timing deviations from the metronomic positions of the metrical grid. Higher levels of beat salience, event density, and fast metrical levels were associated with higher groove ratings, whereas higher levels of MT was associated with lower groove ratings.

These findings are consistent with a functional role of a regular beat as a device for proactive timing that enables synchronization and coordination among individuals. The first three properties that were positively correlated with groove conceivably increase the amount of temporal information, while the fourth and negatively correlated property increases the temporal uncertainty. It stands to reason that fast metrical levels also amount to event density, but not necessarily the other way around, since event density considers any sound events, even those that are not aligned with the metrical grid. Properties related to the music meter, such as the sensation of a regular beat, the beat salience and the metrical levels, have a strong perceptual component in that they are elicited in the listener's mind by the regularities present in the rhythm (e.g., Nozaradan et al., 2011; Honing, 2012). Syncopation is an example of the interaction between the underlying meter and the actual heard rhythm, which manifests itself as a contradiction between the two without allowing for the meter and beat to be challenged (Randel, 1986). To the extent that meter and beat express the temporal expectations of the listener about when musical events will occur, syncopation can be seen as a particular kind of violation of those expectations (Huron, 2006, p. 297). Recently, syncopation has been related to groove perception (Witek et al., 2014).

Micro-timing deviations (MT) result in an expressive variability of the heard durations against a steady and isochronous metrical grid, and in that sense, it can also be considered as an interaction between the physical and perceptual properties of the rhythm. It consists of slightly anticipated or delayed singular sound events (unsystematic MT), repetitive patterns of such deviations (systematic MT), or local changes in the tempo. Typical MT deviations are smaller than what is comprehensibly conveyed by notation, i.e., shorter than the durations that can be considered to be actual metrical subdivisions (<100 ms). MT has now repeatedly been associated with less groove (Butterfield, 2010; Davies et al., 2013; Frühauf et al., 2013). Notably, MT is used in the form of “humanize” functions in commercial music sequencing software and drum machines by applying both systematic and unsystematic timing deviations to quantized temporal events (Hennig et al., 2011). If, as we argue, groove is likely related to synchronization and coordination, Merker (2014) is correct to observe that “[t]he claim that deviations from isochrony constitute the phenomenon of groove or swing is so counter-intuitive as to be tantamount to a contradiction in terms. It asks us to believe that our motivation to engage in predictive synchrony is driven by structural musical content that deviates from, and thus potentially dilutes, obscures, or detracts from, the causal key to that synchrony, which is the isochrony that serves as its predictive basis and target.” In other words, MT is predicted to be negatively associated with groove inasmuch as it decreases synchronization accuracy.

The functional perspective alluded to above is thus consistent with the empirical results obtained so far. It is also with the hypothesis that rhythmic music is a phylogenetic trait favored by selection, as has been more extensively discussed in Madison et al. (2011) and Merker (1999), and with the behavioral and physiological reactions that music in general and rhythmic predictability in particular elicit. For the purpose of the present study, however, it suffices to consider the functional perspective that certain signal properties might facilitate coordination and synchronization, and that the experience of groove may serve to convey the efficacy of an auditory signal for that purpose.

In conclusion, previous research suggests that some general physical properties may underlie groove, but some inherent limitations prevent firm conclusions. While several studies seem to falsify the hypothesis that MT leads to more groove, it may still be effective under conditions that have yet not been experimentally tested. For example, the commercially available music that has been used might rely on other strategies than MT, such as multiple voices and contrasting rhythmical layers, to induce groove. Also, most music was unfamiliar to the listeners, which precludes contextual information. Given the proposed functional purpose of groove-inducing music, at least systematic MT might in fact increase the temporal information if it is related to some performance practice that is shared with the listener. Similar arguments may be raised regarding beat salience, event density, and fast metrical levels. Traditions related to musical styles may direct listeners' attention to certain elements more than others, and the music that has been examined might detract or facilitate some strategies. On a more general level, these uncertainties follow from the study design of previous studies.

Examining pre-existing musical pieces likely yields high ecological validity, but there are two major limitations. Firstly, it is not known to what extent the performers actually intended to induce groove. They may have had quite different artistic goals, at least in a substantial proportion of the music sample. It also leaves open the questions to which extent musicians can intentionally affect groove and what means they use. The music of the pre-existing pieces used in previous research were also ensemble performances, in which different instruments may interact to produce the groove-related signal properties. It is possible that other properties work better to induce groove when there are fewer voices. Secondly, the naturalistic approach is vulnerable to confounding variables. It might be that some musical styles apply certain devices that happen to influence groove, but the reason for using one device rather than another may just be musical tradition and have purely historical causes. Likewise, traditions in music arrangement, choice of instrumentation, and post production might also affect groove.

Here, we take a different approach, and exploit musicians' experience and expressive skills directly by asking them to do whatever they feel is appropriate for increasing and decreasing groove. Being the first study of this kind, it seems appropriate to start by constraining the conditions in order to increase the likelihood of observing similar strategies across performers. This way we may not sample all relevant strategies, but those strategies that are effective within the chosen constraints. Given that groove is related to the fundamental function of synchronization and co-ordination related above, it seems also reasonable that it could be readily conveyed with simple means. Thus, we examine to what extent the intention of a musician to convey more or less groove can be conveyed to a listener by means of expressive performance of a monophonic melody, thereby controlling for possible confounding and confusing effects of multiple musical instruments. We further describe the physical differences between performances that differ in their level of groove, selecting parameters indicated by previous research. Finally, the route from performers' intention to listeners' perception is analyzed as a communication process, using the lens model approach originated by Brunswik (1952, 1956).

We hypothesize that musicians can manipulate perceived groove in melodies played on a keyboard by altering rhythmical and temporal properties of notes, including adding or removing notes within certain constraints. Based on previous research, we also predict that groove be associated with more notes on faster metrical levels, with more notes generally, and with less MT.

## Experiment 1

The following considerations of the design should be noted. First, rhythmical manipulation of a monophonic melody is related to a regular beat, be it explicit or inferred by performer and listener. Second, in order to avoid confusion as to the position of the beat, especially with regard to the performer-listener communication, we made it explicit by adding a simple beat that performers had to synchronize the melodic structure with. This also enables simple and more precise measurement of the timing, as compared to inferring the beat from scattered sound events. Third, given that performers synchronize with the metrical model through an audible signal, we used MIDI information rather than audio, since sequencer programs provide measurement with respect to the metrical structure.

As mentioned in the introduction, rhythmical descriptors like event density and beat salience were found to be correlated with groove in real music examples, whereas MT was negatively or not correlated to groove. We therefore chose to include note onset, offset, and duration data, in terms of positions in the metrical grid, as well as deviations from the grid that were smaller than what could reasonably be considered part of the metrical grid. This type of measure is based on the premise that any kind of MT should be included, that is, we should not apply algorithms that assume systematic patterning or any intention on the part of the musician, and that any type of MT will, however, be reflected in deviations from the grid (Hellmer and Madison, 2014).

### Materials and methods

#### Stimulus production

Four professional male musicians were paid the equivalent of $20 per hour to perform 12 monophonic melodies in various conditions. They were 24–44 years old, could play several instruments, and had a broad experience of different musical styles. The musicians were given recordings of 12 melodies a few days before the recording session. These were deadpan performances without timing or loudness variability, rendered in the sequencer software Cubase 5.0 (Steinberg AG, Hamburg). Melodies which were thought to be unfamiliar to participants were selected, in order to minimize possible extra-musical associations. Six of them (1–6) have a simple rhythmic structure similar to child songs like “Twinkle, twinkle little star” and were composed for this study, because it was difficult to find such melodies that were unknown to the participants. The other six (7–12) are more complex and were adapted from commercially available recordings of jazz and rock style music. The duration of the songs was 16–25 s (*M* = 19.4) and their musical notation is found in Appendix.

Cubase was set up with a default track for each melody, with an accompaniment beat consisting of a kick and hi-hat with constant loudness that sounded simultaneously on every beat and acted as a metronome. The same type of beat was used for every performance, but due to the different structure of the melodies the beat tempi varied across the melodies in the range 120–150 beats per minute (BPM). The musician had to play the melody in synchrony with the beat, which allowed analyzes of its rhythmical properties in relation to the beat and its underlying metrical grid. The musicians played a Yamaha DX7s keyboard connected to a PC via a PreSonus Audiobox USB MIDI interface.

The production session began with the musician recording all 12 melodies in a deadpan version similar to the ones they had listened to. All musicians reproduced the melodies accurately, with the same pitches and canonical note values. After a short break, the musician was asked to perform each melody in two versions, one with the intention of maximizing groove and the other with the intention to minimize groove.

The constraints for the performance were that (1) the melody in the original version had to be recognizable and (2) the same pitches as in the original version had to be used in the same order. Thus, each note could be manipulated in four ways, namely its timing, duration, loudness, and whether its note value was split into more notes with the same pitch or merged with adjacent notes. This was then repeated for all melodies in the orders 1, 7, 2, 8, 3, 9, 4, 10, 5, 11, 6, 12 for two of the musicians, and 12, 6, 11, 5, 10, 4, 9, 3, 8, 2, 7, 1 for the two other musicians. For each melody, the order of the groove conditions was randomized by flipping a coin. The musician was allowed to re-do each condition until he was satisfied with the performance. In total each musician produced 36 performances, 12 deadpan, 12 with groove, and 12 without groove.

#### Design

The independent variables were the type of melody (simple or complex) and groove intention (whether it was performed to minimize or maximize groove). We also include six levels of melody and four levels of performer so as to sample the populations of melodies and performers. The purpose of this was to generalize to these populations, based on the assumption that general tendencies emerge across instances. This constitutes a mixed experimental design with three within-participants (2 melody type × 2 groove intention × 6 melodies) and 1 between-participants (4 musicians) variables, which equals a total of 96 experimental conditions. Although each musician produced 12 deadpan performances, these were not included in the statistical analysis because they were quite accurate and the small performance variability in them could be attributed to unintentional error. Rather, the 12 sequencer-generated versions were used for comparisons of means in the following analyzes, totaling 108 performances.

#### Performance analysis

A number of parameters were calculated from the MIDI data, obtained through the List view in Cubase. This representation of the performance shows the pitch, onset- and offset time, and duration for each sound event. Event density was simply the number of events. Pitch was used to identify the tones in the melody. For each sound event, timing is represented as the position in the measure, defined by the time signature, the position within the beat (1–4), and the number of ticks. This information was used to describe the rhythmic properties of the performance as well as deviations from the canonical metrical structure, i.e., from an idealized deadpan performance. The List view denotes how late a sound event is from the prior 16th note position, rather than how early or late it is from the closest position. Events occurring on 8th note positions were therefore correctly assigned to the closest metrical level by the spreadsheet formula =IF(POS+IF(TICKS>60;1;0)=3;1;0), where POS is the position between 1 and 4 within each beat/quarter note. The time resolution in terms of the number of ticks per beat can be specified in the MIDI protocol, and was in this case set to 480. As the beat constituted quarter notes, the duration of a 16th note is 120 ticks.

Events occurring on 16th note positions were similarly detected by =IF(OR(POS+IF(TICKS>60;1;0)=2; POS+IF(TICKS>60;1;0)=4)=TRUE;1;0). These formulae simply add one 16th note to the prior position if the event is delayed more than one half 16th note (60 ticks). The same formulae were applied to offset positions and note durations, which means that the detected note values were defined by the actual values of performed events, and were unrelated to the notation, which was rather based on the deadpan versions.

The number of ticks that differ from the closest subdivision of 60 ticks constitute the deviation from the closest 16th note, defined as 60 − abs(60 − TICKS), and this unsigned asynchrony from the metrical grid constituted our measure of MT. Because time is defined as a fraction of the beat in the MIDI protocol, one tick corresponds to 60,000/120/480 = 1.04167 ms when the beat tempo is 120 BPM, and 0.83334 ms for 150 BPM. Time deviations are perceptually proportional to the beat however, so the ticks are in fact more relevant than the absolute time, and were used in the subsequent analyzes.

In sum, each note event was assigned a value of 0 or 1 depending on whether it occurred on the relevant metric positions (both 8th and 16th positions), and the parameter used in the following analyzes was the proportion of such events in the performance. The MT parameter was the absolute time difference from the closest 16th note position expressed as a proportion of a 32nd note (TICKS/60). These calculations were also done for event offsets and event durations. The means of these nine values were computed across all events in each performance. Thus, each performance could be characterized by 3 × 3 = 9 parameters plus the number of events. These parameters reflect the amount of syncopation by the fact that syncopation yields more events on fast note values in the same melody.

**Figure 1:**
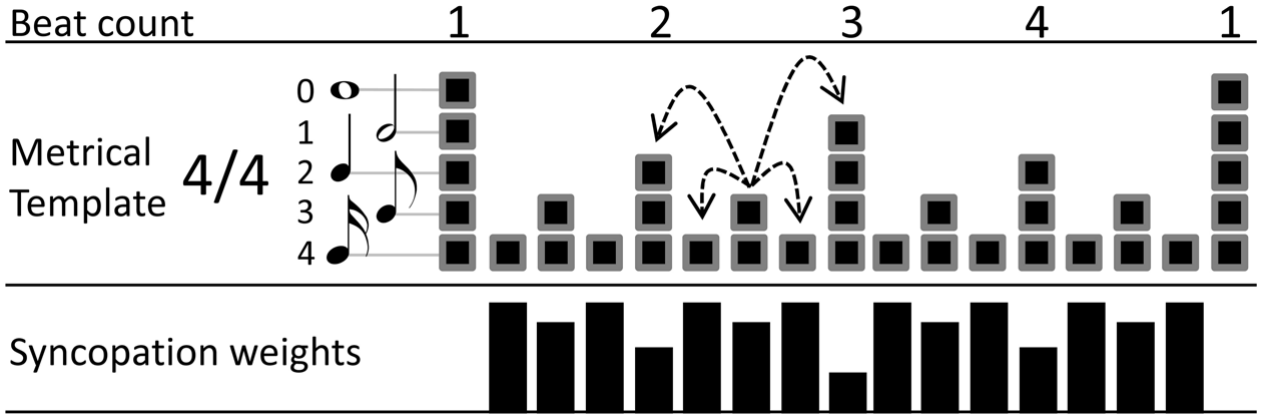
**Example of a 4/4 metrical template used in the SG calculation of the amount of syncopation**. At the bottom, the syncopation weights for each metrical position are shown as black bars. The dashed arrows depict the amplitude differences for the 8th note of the second beat in the bar, as an example of the amplitude differences taken for each metrcial position during the calculation of the algorithm.

In addition, we computed a pure measure of syncopation based on a previously published algorithm (Sioros and Guedes, 2011) (hereafter SG). The SG measure uses a metrical template that associates each metrical position in the bar to the metrical level it initiates to according to the given meter. Each position is assigned a corresponding metrical weight resulting in a pattern similar to the alternating strong and weak beats found commonly in musical meter, e.g., an 8th note position is weaker than a quarter note position and thus it has a lower metrical weight. The metrical template is hierarchical in that each position belongs not only to the metrical level it initiates, but also to all faster ones (Lerdahl and Jackendoff, 1983). Accordingly, an 8th note belongs also to the 16th note level, as seen in Figure 1. Such hierarchical structures represent well the internalized structures of meter in listeners (Palmer and Krumhansl, 1990). The SG algorithm attributes syncopation to the loud notes articulated in weak metrical positions. A loud note articulated at an 8th or 16th note position followed by a quiet note at the quarter note position will be felt as syncopated. The greater the loudness difference the stronger the syncopation. At the same time the positions are not equivalent; the 16th note position syncopation is felt stronger than the 8th note. Therefore, two parameters and their values need to be determined for each note in order to quantify syncopation: (1) how loud the note is and (2) how the metrical strength of its position is translated into a syncopation weight. The syncopation of the note is then calculated as the product of these two factors. Summing the syncopation of all notes in a melody yields the total amount of syncopation.

Notes are considered loud and therefore generating syncopation if they are louder than the notes in their vicinity. In order to quantify how much a note stands out as loud, amplitude differences are taken between that note and the notes in the preceding and following metrical positions. These differences are taken for all metrical levels the note belongs to. As the hierarchical template dictates, a metrical position belongs to the level it initiates and all faster ones. For example, an 8th note position belongs also to the faster 16th note metrical level (Figure 1). Therefore, for an 8th note position, amplitude differences are taken from the preceding and following quarter note positions (the quarter note positions belong also to the faster 8th note level) and the preceding and following 16th note positions (see dashed arrows in Figure 1). Only the onsets of notes are considered and the offsets are ignored. The absence of an onset in a metrical position is equivalent to an onset of zero amplitude, so that amplitude differences are taken even when a position does not carry an actual onset. The average of the two differences—from the preceding and following positions—is calculated for each one of the metrical levels a note belongs to. Subsequently, the lowest of the calculated averages is kept and the rest of the metrical levels are ignored for that note. In that way, it is enough for a note to be preceded and followed by louder notes in one metrical level, to not be considered loud or syncopating. For example an eight note between two quarter notes of equal amplitude will not be considered as a loud note even if the surrounding 16th notes are quiet or absent.

The above algorithm is based on similar principles as the generative model of David Temperley (2009) in which notes at fast metrical levels are much more likely to appear when they are preceded or followed by notes in a slower metrical level (metrical anchoring). Metrical expectations are closely related to the probability of occurrence of events (Palmer and Krumhansl, 1990), so that the unlikely notes that are not surrounded by notes on slower metrical levels can be thought of as syncopation violating these expectations. Temperley's model differs in two important ways from the measure described here. Besides being a generative model and not a syncopation measure, it does not consider the amplitude of the notes that can be an important source of syncopation in a musical performance. In the original SG algorithm, two additional multiplication factors are used during the calculation of the above amplitude differences. First, the amplitude differences are scaled by a factor proportional to the difference of the metrical weights of the corresponding positions. Second, a greater weight is given to the difference from the following metrical position than from the preceding one. The details of the two weights and their values are described and determined in Sioros and Guedes (2011). In the analysis of the simple melodies of the current experiment, these factors as well as all other parameters have been left to their default values (0.5 and 80%).

The second of the two quantities needed in order to calculate the syncopation, the syncopation weight for each metrical position, is calculated according to:

$$S_{i}=1\;-\;0.5^{Li}$$

where *i* indicates the metrical position, *S_i_* is the syncopation potential in the range between 0 and 1, and *L_i_* is the metrical level index starting with 0 for the slowest metrical level and incrementing by one for each faster subdivision (Figure 1). The syncopation potentials are essentially the metrical weights inverted, so that weak positions in fast metrical levels have higher syncopation weight. Finally, the syncopation for each note is calculated as the product of the least average amplitude difference calculated above and the syncopation potential for the corresponding metrical position. The syncopation of the melody is the sum of the syncopation values of all notes.

A comparison of the SG syncopation measure to the more frequently used algorithm proposed by Longuet-Higgins and Lee (1984) (hereafter LHL) has shown that the two measures are in good agreement (Sioros et al., 2012). Although the LHL measure has been tested and compared against human judgments (Fitch and Rosenfeld, 2007; Gómez et al., 2007) while the SG has not, the SG measure was preferred in the current analysis as it considers the dynamic and loudness variations in the performances instead of the simpler binary representation of rhythmic patterns used in the LHL.

The algorithm was originally implemented in the programming language C as a Max/MSP^1^ external that can be downloaded as part of the “kinetic toolbox” at http://smc.inescporto.pt/kinetic. We additionally developed a Max/MSP application around the external in order to read the MIDI files of the performances, extract all the necessary information (time signature, note positions, and MIDI velocities) and format it according to the requirements of the external.

### Results and discussion

Table 1 lists the properties of each melody, including tempo, number of notes, and the six performance parameters. When faced with a communication task such as inducing more or less groove, musicians likely use several means, as has been found for the communication of emotions (e.g., Juslin, 2001). These means can be more or less similar, and variation among them is therefore likely redundant to some extent. Not to overestimate the effect of any one parameter, we applied a simultaneous multiple regression analysis (MRA) model with the groove intention as dependent variable (DV) and the parameters as predictors or independent variables. Although this is backward to the typical MRA application, where the predictors correspond to the independent variables, it is statistically sound because the MRA computationally makes no difference between dependent and independent variables. MRA was therefore used to determine which parameters were affected by groove intention excluding inter-parameter covariance, and their statistical significance was then tested separately. Positive beta weights in Table 2 indicate that higher values (i.e., a greater number of 8th or 16th note events or larger MT deviations) are associated with more groove, since minimum groove was coded 0 and maximum groove was coded 2. Note that the syncopation parameter was not included in the MRA, since it is theoretically redundant to the position parameters. Zero-order point-biserial correlations (*N* = 48) between groove intention and syncopation were 0.30 (*p* < 0.05) for simple melodies and 0.27 (n.s.) for complex ones.

**Table 1:** **Descriptive statistics and performance parameters for the deadpan versions of each melody**.

		**Tempo**	**Number of notes**	**Syncopation**	**Onset 8th**	**Onset 16th**	**Offset 8th**	**Offset 16th**	**Duration 8th**	**Duration 16th**
Simple	1	120	27	0	0	0	0	0	0	0
	2	120	36	0	0	0	0	0	0	0
	3	120	29	0	0	0	0	0	0	0
	4	130	34	0.42	0.147	0	0.147	0	0	0.294
	5	135	29	0.18	0	0	0	0	0	0
	6	130	24	0.25	0	0	0	0	0	0
Complex	7	130	55	8.84	0.527	0.036	0.345	0.509	0.163	0.345
	8	130	35	4.23	0.381	0.143	0.333	0.190	0	0.571
	9	130	28	5.90	0.666	0	0	0.704	0.148	0.296
	10	130	35	2.20	0.193	0	0.158	0.140	0.175	0.386
	11	130	45	3.06	0.432	0	0.704	0	0	0.863
	12	150	45	16.41	0.333	0.422	0.133	0.555	0.089	0.733

*Micro-timing was 0 for all deadpan performances and is therefore not shown*.

**Table 2:** **Beta weights and variance explained from multiple regression analysis for each melody type, expressing the size and direction of each parameters' correlation with the intention to maximize groove**.

**Performance parameter**	**Melody type**	
	**Simple**	**Complex**
8th note onset	**0.57**	**0.49**
16th note onset	**0.38**	**0.54**
Micro-timing onset	0.14	−0.12
8th note offset	**0.48**	0.09
16th note offset	0.23	0.26
Micro-timing offset	−0.09	−0.02
8th note duration	**−0.37**	**−0.35**
16th note duration	−0.17	−0.23
Micro-timing duration	−0.05	−0.08
Density	**0.51**	**0.38**
*R*^2^ (variance explained)	0.562	0.532

*Significant correlations appear as bold (p < 0.05)*.

**Figure 2:**
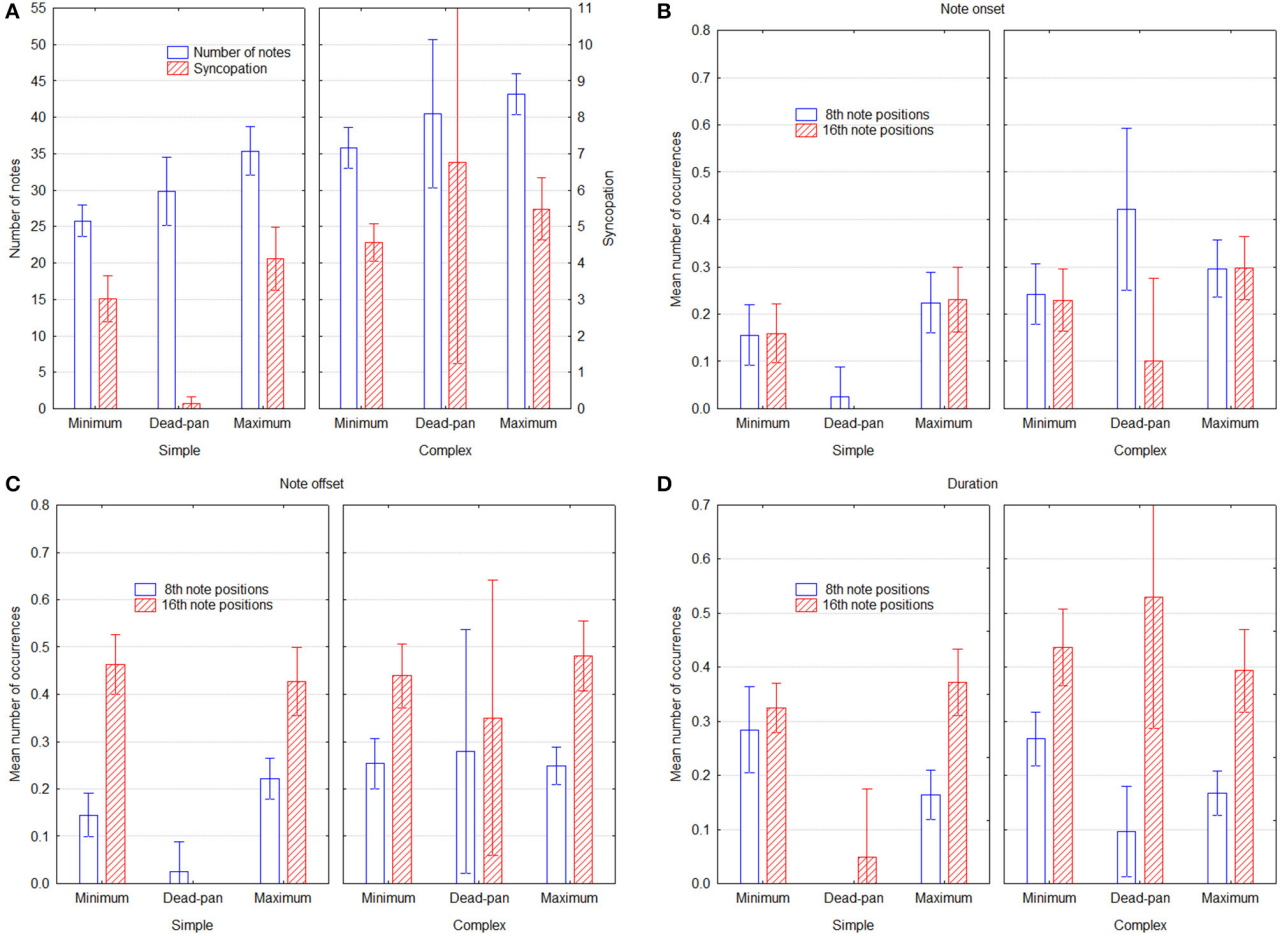
**Performance parameters as a function of groove condition and melody type, across performers**. Panel **(A)** shows the number of notes and the SG syncopation estimate, Panels **(B,C)** show the mean proportion of events that started and ended on 8th and 16th note positions, respectively, and Panel **(D)** shows the mean proportion of 8th and 16th note event durations. Error bars depict 0.95% confidence intervals.

The MRA results suggest a pattern in which groove is associated with higher density, more 8th- and 16th note onsets and offsets, and with fewer 8th- and 16th note durations. MT yields no significant weights at all, but five out of six weights are slightly negative. These patterns are largely the same for simple and complex melodies, but there are two differences. First, 8th note offsets play a greater role for simple melodies than for complex ones. Second, the importance of 8th and 16th onsets seems to be reversed, so that the former is more important for simple and the latter more important for complex melodies. This makes sense because the complex melodies have in fact more fast note values, and that would require adding even smaller note values to increase the metrical levels. These results do not inform about whether musicians increase or decrease these parameters relative to the deadpan versions, however, so the relevant parameters are plotted for all three conditions in Figure 2, Significant differences in Figure 2 can be gleaned from the confidence intervals depicted by the error bars. Panel **A** shows that both the number of notes and the syncopation measure are greater in the maximum groove condition. That syncopation is greatest for the deadpan version is because melody 12 has an extremely high rating (see Table 1), owing to a very complex time structure, and suggests that performers chose not to reproduce this subtle triplet pattern in the maximum and minimum groove conditions. Fewer notes in the minimum condition shows that adjacent same-pitch notes were aggregated given the performance rules above, whereas notes were added in the maximum condition. Apparently performers found additional notes, or the richer rhythmical structure facilitated with them, to be associated with groove. This is also reflected in the syncopation values. The higher values for 8th notes in the deadpan versions of the complex melodies, as seen in Panel **B**, derives mainly from melodies 7 and 9, which are both characterized by a strong back-beat, which the performers apparently decided not to reproduce in its entirety. Otherwise the values for both 8th and 16th onset positions also follow the trend to be higher for maximum than for minimum groove, but here, for the 16th notes, the deadpan versions are much lower. The offset positions in Panel **C** exhibit no difference across groove intention, except for 8th notes in the simple melodies. The duration values in Panel **D**, finally, exhibit no difference for 16th notes, but substantially less 8th note durations for the maximum groove condition. Considering the pattern of MRA weights in Table 2, which is positive for onset and offset and negative for duration, this could be an artifact, in that the same onset and offset position leads to an even duration even if the onset is on an uneven position. For example, an onset one 16th before the beat and an offset a 16th before the next beat will yield an even 4th note duration, and therefore cancel each other out.

## Experiment 2

Experiment 1 manipulated the intention to play with more and less groove and resulted in performances from which rhythmic parameters were measured. Experiment 2 is conceptually the reverse of this, in that we examine the extent to which the manipulations of the performances had the intended effects. First we examine the direct effect of the intention upon perceived groove, and then we combine data from the two experiments through a so-called lens model to examine how the performance parameters were used by performers to convey groove and by listeners to detect groove.

### Materials and methods

#### Participants

Thirty persons were recruited through advertisement on the Umeå University campus, and did not receive any remuneration for their participation. They were 21–45 years old, 12 of them were female, and reported having normal vision and hearing. None of them played music professionally, and were hence regarded as non-musicians. They were randomly divided into two groups with 15 participants, which each listened to the performances of two out of the four musicians.

### Materials

The stimuli consisted of the 108 performances obtained in Experiment 1, 12 of which were deadpan renditions of the 12 melodies, and the remaining 96 performances by the musicians (12 melodies × 2 versions × 4 musicians). All performances featured the same simultaneous kick and hi-hat beat that the musicians heard, in order to provide an unambiguous metric reference and the same conditions that the musicians had. Listener ratings were obtained by a custom made software that played the music examples through the computer sound card and collected the responses. Each listener rated 48 performances from two musicians plus the 12 deadpan versions.

#### Rating scales

In order to minimize possible response bias, the main purpose of the experiment was not disclosed to the listeners, who were instead told that the study was about rhythm perception generally. This was the main reason why the rating scales also included how much they liked the music and how familiar it was to them. In response to the global question “How well do the following terms describe your experience of the music?” and the terms “bekant” (familiar), “bra” (likeable), and “rörelseskapande” (movement inducing) participants entered ratings ranging from 0 to indicate “not at all” up to 10 to indicate “entirely.” Movement inducing was defined in the instructions as “the sensation of wanting to move some part of your body in relation to some aspect of the music.” The ratings were entered through a horizontal slider on the computer screen that could be moved with arrow keys or the mouse, and whose initial default position was set to 5.

#### Procedure

Each participant made the ratings individually, seated in front of a computer in a quiet room and listening through headphones. The participant read a paper with instructions, including the definitions of each rating scale given above, and was asked if the task and terminology was clear. After signing the informed consent form, the participant underwent a training session in which three music examples were rated, different from the ones in the experiment proper, set the volume to a comfortable level, and became familiarized with the program and the procedure. During the experiment proper, which comprised 60 (2 musicians × 2 groove intention + 1 deadpan × 12 melodies) music examples, the participant was left alone in the room to avoid distraction. Upon completion of the experiment the participants were asked about their experience including how difficult it was, if they were tired, and if they felt comfortable with their performance. The entire session took 30–40 min. During the session participants controlled the pace by clicking a button to proceed to the next example. The sliders were blocked while the music was playing, to force them to listen to the whole stimulus before rating.

#### Design

The independent variables were the same as in Experiment 1, but the DV was the rating of perceived groove. It would have been too demanding to rate all 108 performances, so they were divided such that one listener groups rated the deadpan performances and all performances of two musicians, and the other did the same but for the other two musicians. This constitutes a mixed experimental design with 3 within participants variables (2 melody type × 2 groove intention × 6 melodies × 2 musicians (= 48 conditions) and 1 between participants variable (2 listener group), which adds up to 48 × 2 = 96 conditions. In addition to this were the 12 deadpan performances included as a comparison. The conditions were given in five blocks, starting with the deadpan versions, and then two blocks for each musician balanced according to ABBA. Within each block were the melodies were presented in the same random order, but with groove intention randomly varied, which was balanced by the opposite intention in the other block for the same musician. For example, if the melodies are numbered 1 through 12 for simplicity, they occurred in the second block in the order −9, +11, −1, +7, +6, −12, +10, −5, −8, +4, +3, −2, where—denotes minimum groove and + maximum groove. Consequently, they appear in the fifth block (the 2nd A in ABBA) with reversed groove intention, that is, +9, −11, +1, −7, −6, +12, −10, +5, +8, −4, −3, +2. The order in each B block was the same. The purpose of this presentation order was to dilute order effects across both melodies and musicians while at the same time balancing out possible large-scale effects such as fatigue.

### Results and discussion

**Figure 3:**
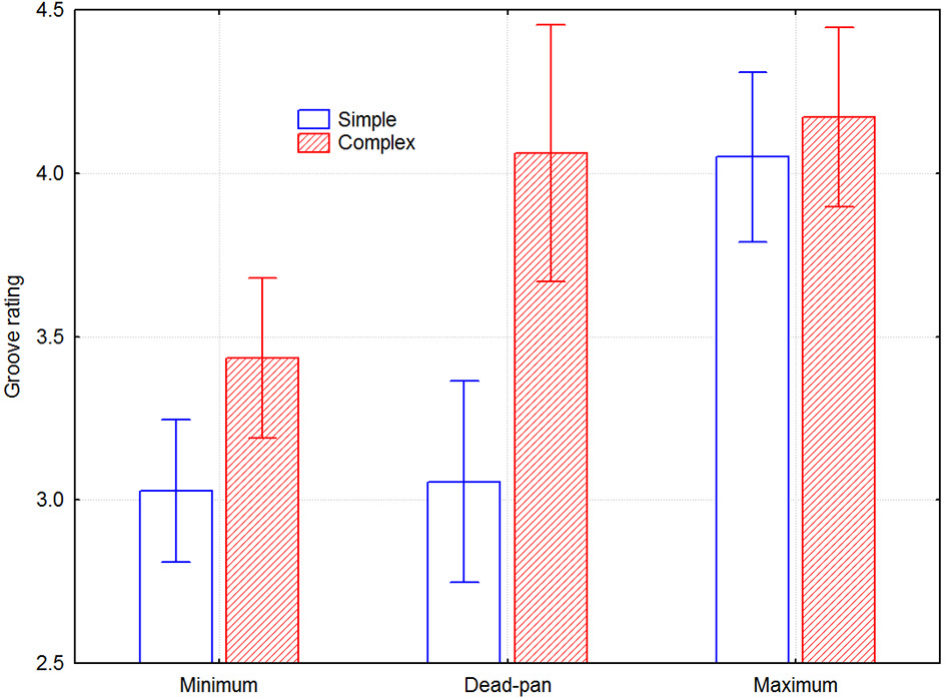
**Perceived groove as a function of groove condition and melody type, across listeners**. Error bars depict 0.95% confidence intervals.

The interviews indicated that all participants understood the instructions, felt comfortable with the task, and did not find it difficult or tiring. The whole design was subjected to a mixed model 2 groove intention × 2 melody type × 6 melodies × 2 musician × 2 listener group five-way ANOVA. There were main effects of groove intention [*F*_(1, 28)_ = 47.06, *p* < 0.000001], melody type [*F*_(1, 28)_ = 25.07, *p* < 0.05], and melody [*F*_(5, 140)_ = 15.03, *p* < 0.000001], but not of musician or listener group [*F*_(1, 28)_ = 0.011, *p* = 0.92; *F*_(1, 28)_ = 2.27, *p* = 0.14]. There were significant 2-way interactions for groove intention × melody type [*F*_(1, 28)_ = 5.54, *p* < 0.05], melody × melody type [*F*_(5, 140)_ = 15.62, *p* < 0.000001], and musician × listener group [*F*_(1, 28)_ = 15.62, *p* < 0.000001]. Since neither melody nor musician number relate to any meaningful dimension, all effects including any of these variables are ignored in the following analysis. They include four significant and six non-significant three-way interactions and four non-significant four-way interactions. Melody and musician number were included in the ANOVA model because possible effects would then be accounted for and hence reduce the amount of unexplained variance. Figure 3 depicts the remaining effects of interest, namely groove intention and melody type. The effect size of the groove intention was 0.31 for the simple melodies and 0.21 for the complex melodies.

## Lens model analysis across experiments

The so-called lens model is suitable for analyzing communication processes such as the present one, in which an intended message may be conveyed through multiple cues that may be combined to interpret the message by the receiver (Brunswik, 1952, 1956). The lens model can be visualized as two folding fans with their respective rays at the far ends facing each other. The proximal end of the left fan represents the sender, in this case the intention to induce groove or not, and the proximal end of the right fan represents the receiver, that is, the listeners' perception of groove. The rays that extend from each endpoint represent the multiple cues that are partly embodied in the sounding performances, which are represented by the lens situated in the intersection of the rays from both fans. The cues are in this case the parameters that we have selected, but could potentially be any other property that was not selected. The right-hand fan similarly represents how the cues are used by the listeners to arrive at their experience of groove. Several outcomes are thus possible. First, the cues—i.e., the parameters that were measured—might explain more than the direct effect of the intention upon the level of perceived groove. This could happen if these cues are used differently by performers and listeners, for example if performers change cues that the listeners do not respond to, and vice versa. Second, and more likely, the cues might explain less than the direct effect of the intention upon perceived groove. This would be the case if not all relevant cues were included in the model.

The lens model equation (LME) expresses the first-order correlation between the sender and receiver as a function of a matching factor G, the multiple correlations of the regression models for senders (R_s_) and receivers (R_r_), respectively, and an un-modeled component that consists of the correlation between the residuals in the regression models C and the residual variation of the models (Hursch et al., 1964):

(1) $$r_{a}=\text{G }{\text{R}}_{\text{s}}{\text{R}}_{\text{r}}+C\;\sqrt{\left(1-{\text{R}}_{\text{s}}^{2}\right)}\sqrt{\left(1-{\text{R}}_{\text{r}}^{2}\right)}$$

G is calculated as the correlation between the predicted values for the sender and receiver regression models, and C is likewise the correlation between the residual values for the sender and receiver regression models. With proper correction for the unexplained variance in the second term of Equation 1, the achievement (*r_a_*) then equals the simple (point-biserial) correlation between groove intention and groove ratings. The lens model factors are summarized in Table 3. These indices reflect relatively successful communication for the simple melodies, but poor for the complex ones. G is quite high for the simple melodies, indicating that performers and listeners are rather well matched to each other, that is, that they share a common code. The two R factors indicate that the cues are not used fully consistently, in particular not by the listeners. For the complex melodies it is primarily poor matching that limits the communication, because the consistency in cue utilization by the performers is as high as for the simple melodies, but somewhat lower for the listeners. Although it is hard to derive this from the regression models, listening to the performances suggests that performers in the minimize condition sometimes simplify the melodies and make them very quantized, and sometimes play erratically so as to destroy the beat altogether. It is obvious that both these strategies lead to less groove, since they both decrease temporal information, but they do so in quite different ways. This is probably the reason for the low matching.

**Table 3:** **Lens model factors for the same performance parameters as in Table 2, for simple, complex, and both types of melodies**.

	**Melody type**	*****r***_**a**_**	**G**	**R_**s**_**	**R_**r**_**
Performance parameters	Simple	0.620	0.83	0.749	0.687
	Complex	0.394	0.34	0.729	0.523
	Both	0.489	0.78	0.621	0.490
Syncopation	Simple			0.305	0.205
	Complex			0.270	−0.103
	Both			0.273	0.094

*The lower part lists the zero-order correlations for the syncopation parameter. Note. C was 0.40 ± 0.04*.

The un-modeled component of the communicative process includes both unsystematic and systematic variance not accounted for by the linear models. This includes effects of inconsistency in cue utilization, order effects, distractions, memory intrusions, omission of relevant cues, etc. If C is high, it may indicate (a) a common reliance on cues not included in the regression models, (b) chance agreement between random model errors, (c) cue interactions common to both models, or (d) nonlinear cue function forms common to both models (Cooksey, 1996). That C was low (0.37–0.44) indicates that we have not excluded cues that performers and listeners would have used consistently.

The differences between simple and complex melodies seen in Figure 2 suggest that there might be different patterns in the multiple regression beta weights. However, the model was not run separately for the types of melodies because that would mean having too few cases (48), in particular in relation to the nine predictors (e.g., Kelley and Maxwell, 2003).

## General discussion

The main purpose of the present study was to investigate what musicians do to increase and decrease groove and validate to what extent these strategies are effective, according to listeners' ratings. The results demonstrates that groove is conveyed by simple means, including syncopation. For the simple melodies, the musicians' intention explained 56 percent of the variance in the performance parameters, which in turn explained 47 percent of the variance in the listeners' ratings (the squared r_s_ and r_r_ values in Table 3). Because the model matching was less than perfect, the overall achievement was less than 50 percent. This must be considered a very good level of communication, given that the listeners were lay people, and that the measurement of both parameters and perceived groove are associated with measurement error. It was considerably less successful for the complex melodies, owing to the fact that they were rated as having the maximum level of groove found in the study already in their deadpan versions. Therefore, most of the communication in their case consisted of decreasing groove. The smaller effects for the complex melodies indicate that it was much more difficult to decrease groove in already rhythmically complex structures than to increase groove in the simple structures.

We also found that groove could intentionally be reduced in two different ways. These ways were only indirectly tapped into by the multiple regression models, but required listening to the music performances to become apparent. One strategy was to simplify the rhythmic structure of the melodies by moving events from faster metrical levels to slower ones, which inevitably leads also to a decrease in syncopation. The other was to play quite erratically so as to break up the sense of a steady pulse, although it is unclear how this should be formally described. Such strategies might lead to both higher values in the measurements of MT deviations and higher numbers of events in fast metrical levels. The latter is, however, an artifact of the tools used in the analysis, which requires a metrical context and assumes that this metrical context is correctly represented by the MIDI time signature. Clearly, this is not the case when the intention of the musicians is to challenge instead of support the imposed beat. A sophisticated measure such as the SG syncopation index might therefore report large values that are not representative of the actual musical qualities, because it has no way of knowing that the musician's intention is to “damage” the pulse. On the one hand, it is therefore quite difficult to discern between these strategies and it cannot be done with the present simple rhythm indicators. On the other hand, these are extreme strategies that are unlikely to be employed in real performances. This sophistication may also be one reason why the SG measure explained less variance, as it may be more sensitive to differences between musically well-formed rhythms than to coarse differences including both deadpan and intentionally poor performances.

Thus, all the strategies by which musicians successfully manipulate perceived groove are consistent with the function of enabling and facilitating entrainment amongst individuals (Madison et al., 2011; Merker, 2014). This is based on the premise that a stable beat with less variability and distractions (Davies et al., 2013), as well as with richer temporal information (Madison, 2014) should facilitate synchronization and co-ordination.

The reasons why MT has nevertheless been so frequently discussed as a physical cause of groove are historical, and seem to be mainly related to three circumstances. The first is that many musicians state that they play certain notes early or late, as heard in many interviews, the second is that music performances factually exhibit considerable variability from the canonical metrical structure, and the third is that an entire book and a few other influential articles claimed this was the case (reviewed in Iyer, 2002), possibly as a plausible but incorrect interpretation of the two first observations. In reality, it is very difficult to produce or detect certain physical deviations, because that is not what musicians are trained to do—it is rather to achieve a certain perceptual result.

One limitation with the present study is that the relatively open task of the musicians leaves room for confounding variables, which is an inevitable downside of this naturalistic and ecologically valid approach. Another limitation is that there were only six melodies of each kind. Clear differences between the two types of melodies and a high correspondence between performance and listener parameters across melodies both attest to the validity of the results, however. It might still be that the melodies used here are less representative for melodies in general, as they were not randomly sampled from the whole population of melodies. Future research should therefore test the effects of systematically applying syncopation, preferably on different, randomly sampled, and greater numbers of melodies (see Sioros et al., in review).

Groove is a treasured property of much music, such as jazz, reggae, samba, salsa, and many more styles. Within these idioms, music groups and even individual musicians are known to produce more or less groove (Schuller, 1989; Iyer, 2002). It is therefore interesting to speculate whether these differences are intentional or have to do with individual differences in ability. In other words, are musicians trying equally hard, but succeed differently? No studies to date seems to have addressed musicians' intentions, but all have used already available music examples, for which the intention was unknown. One unique contribution of the present study is that musicians were actually asked to play with as much and as little groove they could. Another unique contribution is that the whole communicative chain was investigated, whereas previous studies of performance merely assumed that musicians' expressive intentions would be perceived by listeners (Alén, 1995; Prögler, 1995; McGuiness, 2005; Gratier, 2008).

This leads to the issue that musicians' intentions may be related to their understanding of the listeners' musical experience and pre-knowledge, such that their implementation of groove be optimized for various types of listeners. It is conceivable that training increases the acuity of perceptual abilities, such that one becomes better at extracting the pulse in more complex rhythms, for example. At the extreme end of that spectrum, a culture-relativistic view would assume that the perception and appreciation of phenomena relies entirely on enculturation to them. At the other end of that spectrum are human universals that rely on invariants in human milieu and heritable traits.

A few studies have considered individuals' music or dance experience, which seems to lead to a somewhat higher sensitivity and appreciation for groove. Differences are marginal, however, and suggest slight modulation of the same system, rather than any qualitative difference (Witek et al., 2014). A new study (Madison et al., in review) shows that individuals are equally apt at gauging groove regardless of how much they like or are familiar with the musical style. Here, the relatively small number of participants precluded analysis of the effects of training.

It is notable that the new insights we have gained from giving musicians relatively free rein provide strong support for the functional theory of groove, since all the strategies applied to increase groove conceivably increase the effectiveness of the signal for precise synchronization and co-ordination, and vice versa (Madison et al., 2011; Davies et al., 2013). Likewise, the increased number of notes on faster metrical levels that the musicians performed with the intention to maximize groove is also known to increase synchronization accuracy (Madison, 2014). The functional theory of groove states, in brief, that synchronization is associated with an adaptive value here and now or in some earlier stage of human evolution (Merker et al., 2009; Madison et al., 2011). One scenario to this effect was proposed by Merker, according to which synchronous vocal exclamations reach farther and are thus heard by a larger number of conspecifics (Merker, 1999). Synchronization is characterized by predictive timing rather than reaction, and requires therefore a good internal temporal model. This model is enhanced by rich temporal information from the external signal that is used to guide synchronized or coordinated movement (Madison et al., 2011; Merker, 2014), and a behavioral tendency toward synchronization in general and an appreciation of signal properties that enhance it would therefore have increased in the population.

In conclusion, the present study extends previous findings that event density and fast metrical levels are associated with groove, according to listener ratings, by demonstrating that musicians actually use them to convey groove. Our hypotheses were supported, and we show in addition that syncopation is a central strategy for inducing groove, and suggest types and frequencies of syncopes that should be particularly effective (Sioros et al., in review). A closer examination of the relation between syncopation and groove may be informative of cognitive mechanisms related to the perception of complex rhythmic structures.

## Author contributions

Conceived and designed the experiment: Guy Madison. Performed the experiment: Guy Madison. Analyzed the data: Guy Madison, George Sioros. Contributed analysis tools: George Sioros. Wrote the paper: Guy Madison, George Sioros.

### Conflict of interest statement

The authors declare that the research was conducted in the absence of any commercial or financial relationships that could be construed as a potential conflict of interest.

## Footnotes

^1^www.cycling74.com
